# Artificial Intelligence in Chronic Lymphocytic Leukemia: From Prognostic Biomarkers to Individualized Treatment Selection

**DOI:** 10.3390/diagnostics16213523

**Published:** 2026-10-27

**Authors:** Bogdana Ghimpău, Sandra Timar, Dacian Nicolae Oros, Alexia Ioniță, Despina Calamar-Popovici, Ramona Cioacă, Ioana Ioniță

**Affiliations:** 1 Doctoral School of Medicine, “Victor Babes” University of Medicine and Pharmacy, 300041 Timisoara, Romania; bogdana.cioaca@umft.ro; 2 Department of Allergology and Clinical Immunology, “Pius Brinzeu” Emergency Hospital, 300723 Timisoara, Romania; 3 First Department of Internal Medicine, “Victor Babes” University of Medicine and Pharmacy, 300041 Timisoara, Romania; sandra.lazar@umft.ro (S.T.); oros.dacian@umft.ro (D.N.O.); despina.calamar.popovici@umft.ro (D.C.-P.); ionita.ioana@umft.ro (I.I.); 4 Multidisciplinary Research Center for Malignant Hematological Diseases (CCMHM), “Victor Babes” University of Medicine and Pharmacy, 300041 Timisoara, Romania; 5 Faculty of Medicine, “Victor Babes” University of Medicine and Pharmacy, 300041 Timisoara, Romania; alexia.ionita@student.umft.ro; 6 Department III—Functional Sciences, “Victor Babes” University of Medicine and Pharmacy, 300041 Timisoara, Romania

**Keywords:** chronic lymphocytic leukemia, artificial intelligence, evidence map, conditional average treatment effect, causal inference, treatment selection, shared decision-making

## Abstract

Available artificial intelligence (AI) applications in chronic lymphocytic leukemia (CLL) mainly address diagnosis, prognosis, treatment need, or measurable residual disease rather than comparative treatment selection. This state-of-the-art review examines the gap and proposes a CLL-specific clinical decision-support architecture. A focused evidence map was assembled by title-and-abstract screening of 245 unique PubMed records retrieved using a documented 2014–September 2026 search strategy. Fifty-five original CLL-relevant AI and machine learning (ML) reports were retained for descriptive mapping. Existing tools are heterogeneous and rarely establish comparative causal effects under competing regimens. We define individualized selection using clinically eligible treatment sets, explicit treatment contrasts, regimen-specific outcomes, calibration and uncertainty checks, and post hoc longitudinal patient-preference filtering. The architecture is conceptual, not a trained or externally validated treatment-selection algorithm; prospective studies will be necessary to demonstrate clinical utility.

## 1. Introduction

The therapeutic landscape of chronic lymphocytic leukemia (CLL) has changed profoundly. Chemoimmunotherapy has largely been displaced by targeted therapy, including continuous Bruton tyrosine kinase (BTK) inhibition, fixed-duration B-cell lymphoma 2 (BCL2) inhibitor-based therapy, fixed-duration BTK inhibitor–BCL2 inhibitor combinations and in selected later-line settings, noncovalent BTK inhibition. The 2026 European Hematology Association (EHA) guideline explicitly frames CLL management as a holistic process in which disease-specific and patient-specific criteria interact throughout decision-making [1]. The 2024 Lymphoma Research Foundation consensus similarly emphasizes molecular profile, comorbidities, concomitant medications, toxicity, treatment duration and patient preference when selecting and sequencing therapy [2].

This progress has also changed the clinical problem. For many patients, the issue is no longer whether an effective treatment exists. It is which of several effective options makes the most sense for that particular individual. Clinical staging and composite prognostic indices remain useful [3,4], and molecular characterization has sharpened risk assessment. TP53 (tumor protein p53 gene) disruption and immunoglobulin heavy-chain variable region (IGHV) mutational status are still central. However, they are now interpreted in a therapeutic landscape that is far more varied than the one in which many traditional prognostic tools were developed.

Treatment selection therefore depends not only on disease biology, but also on the characteristics and priorities of the individual patient. The same biological profile can lead to very different treatment discussions in a patient with recurrent atrial fibrillation on anticoagulation, another with chronic kidney disease and bulky lymphadenopathy, or a third with recurrent respiratory infections and hypogammaglobulinemia. Preferences matter as well. A fit, professionally active patient who strongly values a treatment-free interval may view continuous and fixed-duration therapy quite differently from a frail patient for whom intensive early monitoring is itself a burden.

Artificial intelligence (AI) and machine learning (ML) may be particularly useful in addressing this multidimensional decision problem. CLL already offers proof of concept. The CLL Treatment–Infection Model (CLL-TIM), developed from 4149 patients and an ensemble of 28 algorithms, predicted severe infection and/or treatment within two years while also providing individualized risk factors and estimates of uncertainty [5]. Other groups have built explainable models from routine laboratory and demographic data to predict treatment need [6], and AI-assisted approaches have improved the efficiency of flow-cytometric measurable residual disease (MRD) assessment [7,8].

Nevertheless, a 2025 systematic review of ML in CLL identified only 20 eligible studies published between 2014 and 2023 and highlighted recurrent limitations including small or single-center datasets, overfitting risk, limited real-world applicability and insufficient external validation [9]. Moreover, most published models address diagnosis, prognosis, treatment need, MRD detection or outcome prediction rather than the comparative treatment question faced by clinicians.

Predicting prognosis is not the same as choosing treatment. A model may accurately estimate progression, survival, or toxicity and still provide little information about which of two reasonable therapies is likely to offer the better balance for an individual patient. This review therefore examines what would be required to move from disease-centered risk prediction toward individualized treatment decision support in CLL. In this context, AI is considered as a tool for integrating diagnostic, prognostic, treatment-specific, and patient-level information to support clinical decision-making rather than to replace clinician judgment. The distinct contribution of this review is an operational, CLL-specific linkage of four previously separated steps: (i) establishing a clinically eligible regimen set, (ii) defining explicit causal contrasts for treatment-specific benefit and harm, (iii) auditing whether these estimates are calibrated and sufficiently supported by comparable patients, and (iv) eliciting evolving patient preferences after, not inside, the clinical risk model. This is a falsifiable design proposal rather than an algorithm with demonstrated predictive or clinical performance [10,11,12,13,14].

## 2. Methods: State-of-the-Art Narrative Review and Focused Evidence Map

This study is a state-of-the-art narrative review with an explicitly delimited, focused evidence map of original AI/machine-learning research pertinent to CLL. The objective is to integrate clinical, laboratory, causal-inference and decision-support concepts, not to estimate a pooled effect or claim an exhaustive systematic synthesis. The defined topical publication window was 1 January 2014–30 September 2026. Unlike a systematic review, independent duplicate screening, systematic full-text risk-of-bias appraisal and quantitative synthesis were not undertaken. The focused map is confined to original patient-, sample- or disease-relevant AI/ML reports concerning CLL diagnosis, molecular stratification, progression, treatment-related outcomes or MRD. Contextual CLL trials, guidelines and methodological standards were synthesized narratively outside this count.

For this revision, the supplied PubMed/MEDLINE export obtained on 20 October 2026 was screened at the title-and-available-abstract level. It contained 245 records with distinct PubMed identifiers (zero within-export duplicates). Fifty-five original CLL-relevant AI/ML reports were retained for the focused descriptive evidence map and 190 were excluded. Exclusion categories comprised reviews or non-original discussion, computational chemistry or mechanistic models without direct relevance to the clinical AI/ML task, unrelated records or ambiguous uses of the acronym CLL, and applications outside the defined focused scope. Each record and its screening decision was logged. Figure 1 reports these real counts and does not imply systematic full-text review. The published systematic overview of earlier CLL ML studies [9] was additionally used for context and citation verification; its prior selection was not counted as a second independent database search.

Using Medical Subject Headings (MeSH) and title/abstract fields, the exact PubMed search comprised (“Leukemia, Lymphocytic, Chronic, B-Cell”[MeSH Terms] OR “chronic lymphocytic leukemia”[Title/Abstract] OR “chronic lymphocytic leukaemia”[Title/Abstract] OR “small lymphocytic lymphoma”[Title/Abstract] OR CLL[Title/Abstract]) AND (“Artificial Intelligence”[MeSH Terms] OR “Machine Learning”[MeSH Terms] OR “Deep Learning”[MeSH Terms] OR “Natural Language Processing”[MeSH Terms] OR “artificial intelligence”[Title/Abstract] OR “machine learning”[Title/Abstract] OR “deep learning”[Title/Abstract] OR “neural network”[Title/Abstract] OR “random forest”[Title/Abstract] OR “support vector machine”[Title/Abstract] OR XGBoost[Title/Abstract] OR “explainable”[Title/Abstract] OR “natural language processing”[Title/Abstract] OR “automated classification”[Title/Abstract] OR “automated analysis”[Title/Abstract] OR “computer-aided”[Title/Abstract] OR “computational”[Title/Abstract] OR “radiomics”[Title/Abstract] OR “digital pathology”[Title/Abstract] OR “predictive model”[Title/Abstract]) AND 2014/01/01:2026/09/30[dp]. No additional language or publication-type filters were applied. Screening was performed on the exported record list; the search date and term syntax are reported to permit rerunning it. Database indexing may change subsequent retrieval counts.

Synthesis was descriptive and thematic rather than statistical. Retained records were mapped by author, publication year, directly studied AI/ML task and principal limitation for comparative treatment selection. Where a numerical estimate, validation procedure or model-calibration detail was not reported in the available abstract, it was not inferred. The additional methodological audit considers sample and event size, data leakage, missing data, internal, external and temporal validation, calibration, optimism correction and clinical utility. Since all primary full texts were not independently re-extracted, this is a reporting-oriented appraisal, not a formal updated Prediction model Risk Of Bias ASsessment Tool for artificial intelligence (PROBAST + AI) assessment [15]. Evidence was interpreted across three distinct questions: prognosis or need for treatment; outcomes under observed therapy; and counterfactual comparative treatment effects [10,11]. This separation is central to the state-of-the-art rationale.

ChatGPT (OpenAI, GPT-5.6 Sol) was used during manuscript preparation for editorial restructuring, language editing, and conceptual figure layouts. It was not used to generate clinical data, perform patient-level analyses, train or validate a predictive model, or derive treatment-effect estimates. The authors remain responsible for scientific interpretations, bibliographic accuracy, and approval of the submitted manuscript.

## 3. From Prognostic Stratification to Individualized Treatment Selection

Traditional prognostic systems were developed primarily to estimate disease course. Rai and Binet staging remain clinically useful, while the CLL International Prognostic Index (CLL-IPI) integrates age, clinical stage, β2-microglobulin, IGHV mutational status, and TP53 disruption [4]. The International Workshop on Chronic Lymphocytic Leukemia (iwCLL) criteria provide a standardized framework for diagnosis, indications for treatment, and response assessment [3]. These tools address essential clinical questions, but they were not designed to estimate the comparative benefit of multiple modern targeted therapies for an individual patient. This review uses “individualized treatment selection” to mean the comparison, at a defined CLL clinical decision time, of the expected outcome distribution under each eligible regimen for a patient with covariates X, followed by transparent discussion of trade-offs. The clinically eligible set is established from guidelines, previous therapy, contraindications, access and patient circumstances before risk prediction; a high model score never overrides an established absolute contraindication [1,2].

Prognostic and predictive factors are related but not interchangeable. A prognostic factor is associated with outcome independently of the treatment comparison under consideration, whereas a predictive marker modifies the relative effect of one treatment compared with another. In clinical practice, this distinction is not always maintained. TP53 disruption has clear therapeutic relevance, but renal function, anticoagulation, infection history, frailty, and treatment preference represent different types of information. They influence the expected consequences, feasibility, or acceptability of a treatment even when they are not validated predictive biomarkers. Table 1 summarizes the main biological and patient-related factors that may contribute to individualized treatment selection in CLL, together with their established roles and important limitations [16].

The question at the bedside is therefore broader than “How aggressive is this patient’s CLL likely to be?” Clinicians increasingly need to compare several reasonable treatment strategies for the same individual. Disease control remains central, but so do toxicity, treatment burden, feasibility, and preservation of future treatment options. Progression-free survival, important as it is, cannot capture the entire decision [1,2,16].

This distinction has methodological consequences. If an AI model trained to predict outcomes is used to recommend therapy without explicitly estimating treatment effects, a prognostic association may be misinterpreted as evidence that one treatment is preferable to another. This becomes particularly important when predictive models developed for risk estimation are extended into therapeutic decision support [10,11].

## 4. Contemporary CLL Therapy: When Efficacy Is Not the Only Consideration

Modern first-line CLL therapy includes several highly effective targeted options. Long-term follow-up of CLL14 demonstrated durable benefit after one year of fixed-duration venetoclax–obinutuzumab in patients with coexisting conditions, with a median progression-free survival (PFS) of 76.2 months and an estimated six-year time to next treatment of 65.2% [17]. Fixed-duration BTK inhibitor–venetoclax combinations have also produced deep responses in CAPTIVATE and GLOW [18,19], while the FLAIR platform has provided randomized evidence for MRD-directed ibrutinib–venetoclax [20,21].

The phase 3 AMPLIFY trial further expanded the fixed-duration treatment landscape by demonstrating improved PFS with acalabrutinib–venetoclax, with or without obinutuzumab, compared with chemoimmunotherapy in previously untreated CLL without del(17p) or TP53 mutation [22]. The 2026 EHA guideline consequently includes acalabrutinib–venetoclax, with or without obinutuzumab, among contemporary time-limited treatment options [1].

CLL17 is particularly informative because it directly compares fixed-duration and continuous targeted treatment. Among 909 previously untreated patients, three-year PFS was 81.1% with venetoclax–obinutuzumab, 79.4% with ibrutinib–venetoclax, and 81.0% with continuous ibrutinib; both fixed-duration regimens met the prespecified criterion for noninferiority [23]. Following treatment, undetectable MRD in peripheral blood was observed in 73.3%, 47.2%, and 0% of patients, respectively [23]. Long-term data also support the durability of established targeted strategies. Five-year follow-up from SEQUOIA showed sustained disease control with zanubrutinib in treatment-naive CLL/small lymphocytic lymphoma (SLL) [24], while the final MURANO analysis confirmed durable benefit from fixed-duration venetoclax–rituximab in relapsed/refractory disease [25].

These results do not imply that contemporary targeted regimens are clinically interchangeable. Rather, when several strategies provide effective disease control, differences in treatment duration, toxicity profile, monitoring requirements, comorbidities, and patient preferences become increasingly relevant to treatment selection. The decision therefore extends beyond efficacy alone and requires consideration of the balance between expected benefit, treatment-related risk, and treatment burden for the individual patient [1,2,16].

Continuous BTK inhibitor therapy avoids the ramp-up and tumor lysis syndrome monitoring required for venetoclax initiation, but it involves prolonged drug exposure, sustained adherence, and the potential for cumulative toxicity. Fixed-duration venetoclax-based therapy provides a defined treatment end point but may require more intensive monitoring during initiation. BTK inhibitor–BCL2 inhibitor combinations introduce a different balance of treatment duration, response depth, toxicity, and monitoring requirements. The principal clinical considerations associated with these strategies are summarized in Table 2 [2,16].

In particular, the eligible comparison changes with line of therapy, TP53 disruption, previous exposure, tolerance, resistance, and availability. A treatment-ranking model cannot legitimately transport first-line comparative estimates to double-exposed relapsed disease. Table 3 lays out distinct target decisions; the alternatives are illustrative and conditional upon current guidelines, licensing, local availability, fitness, drug interactions, and resistance [1,2,16,22,23,24,25].

## 5. Toxicity as a Factor in Choosing Treatment

### 5.1. Cardiovascular Vulnerability and BTK Inhibition

Cardiovascular toxicity provides a clear example of the interaction between treatment characteristics and patient vulnerability. International consensus recommendations support baseline cardiovascular assessment before BTK inhibitor therapy and recognize that more selective BTK inhibitors may be preferred in patients with increased cardiovascular risk [26]. Importantly, BTK inhibitors should not be considered a homogeneous class.

In the final comparative analysis of ALPINE in relapsed/refractory CLL/SLL, atrial fibrillation (AF) or flutter occurred in 7.1% of patients receiving zanubrutinib and 17.0% of those receiving ibrutinib; overall cardiac events occurred in 25.9% and 35.5%, respectively [27]. ELEVATE-RR similarly demonstrated lower rates of atrial fibrillation/flutter, hypertension, and bleeding with acalabrutinib than with ibrutinib in previously treated high-risk CLL [28]. Real-world comparative analyses have broadly supported a more favorable cardiovascular profile for acalabrutinib than for ibrutinib [29]. Treatment-aware machine-learning analyses have also examined AF risk at BTK inhibitor initiation, but such observational predictions should not be conflated with validated regimen-specific comparative treatment effects [30].

These differences are relevant to individualized modeling because representing “BTK inhibitor exposure” as a single treatment variable may obscure clinically meaningful differences between individual agents. Cardiovascular risk assessment should also extend beyond a simple history of atrial fibrillation. Hypertension, heart failure, ischemic heart disease, ventricular arrhythmias, renal impairment, and concomitant cardiovascular medications may each contribute additional information [26,31,32].

### 5.2. Bleeding, Anticoagulation, and Polypharmacy

Bleeding risk is particularly relevant in an older CLL population, in which anticoagulant and antiplatelet therapies are frequently used. Previous bleeding, thrombocytopenia, type of anticoagulant, antiplatelet exposure, renal function, and potentially interacting medications should therefore be captured separately whenever possible. Polypharmacy may further reflect both the potential for drug–drug interactions and the overall complexity of treatment. Reducing these factors to a single comorbidity count may obscure information that is relevant to treatment selection [26,31,32].

### 5.3. Renal Function, Tumor Burden, and Venetoclax

Venetoclax-based therapy introduces a different toxicity and monitoring profile. Tumor burden and renal function are central to TLS risk assessment, and established mitigation strategies include dose ramp-up, hydration, urate-lowering therapy, and laboratory monitoring according to the estimated level of risk [33]. A fixed-duration venetoclax strategy may therefore be attractive in a patient with substantial cardiovascular vulnerability, while its initiation may be more medically and logistically demanding in a patient with impaired renal function and bulky disease.

### 5.4. Infection and Immune Vulnerability

Infection remains a major source of morbidity in CLL. Baseline immune dysfunction, previous severe or recurrent infections, hypogammaglobulinemia, neutropenia, chronic pulmonary disease, age, and cumulative treatment exposure may all contribute to infectious risk. CLL-TIM demonstrates that infection-related vulnerability can be modeled using routinely available clinical data [5]. However, predicting infection risk and using that prediction to choose between alternative therapies are distinct problems. How predicted infectious risk should influence comparative treatment selection remains insufficiently defined [34].

## 6. Frailty and Functional Reserves as Factors in Choosing Treatment

Chronological age is an inadequate surrogate for physiological reserve. Frailty may influence treatment tolerance, adherence, hospitalization risk, and the practical burden of monitoring. In a prospective cohort of 82 patients aged 65 years or older receiving BTK or BCL2 inhibitors, a Clinical Frailty Scale (CFS) score > 3 was associated with treatment-related toxicity and a higher rate of treatment discontinuation; discrimination for toxicity was moderate (area under the curve (AUC) = 0.695) [35].

These findings support frailty as a clinically relevant component of treatment assessment, but an AUC of approximately 0.70 is insufficient to support its use as a stand-alone treatment selector. Frailty is more informative when considered as part of a broader assessment of patient vulnerability. It is also not necessarily static. Some deficits may be reversible, others persistent, and functional status may change substantially during treatment. A single baseline measurement may therefore fail to capture clinically relevant change over time [35].

## 7. Preferences of Patients in Decision-Making

Patient preferences should be incorporated into treatment selection rather than considered only after a medical decision has been made. In CLL, individual priorities may influence which of several clinically reasonable strategies is most acceptable to a particular patient. In a discrete-choice experiment involving 229 patients, improvement in two-year PFS was highly valued, but fixed-duration treatment, oral administration, and lower risks of adverse effects were also important attributes [36].

The CHOICE study included 401 patients from 16 Italian centers and showed that treatment duration, infection risk, and organ toxicity strongly influenced treatment preferences [37]. Because enrollment occurred during the first wave of the coronavirus disease 2019 (COVID-19) pandemic, the particularly high importance assigned to infection risk should be interpreted within that context rather than generalized to all treatment settings.

A multinational study published in 2025, involving 192 patients and 259 physicians, found that five-year PFS was the most important treatment attribute for both groups, followed by common side effects and adverse events leading to treatment discontinuation; patients also expressed a strong preference for time-limited therapy [38]. In 2026, a US study involving 150 patients and 150 oncologists identified distinct preference patterns that were not adequately explained by observed disease characteristics or sociodemographic variables [39].

For AI design, the implication is straightforward: ask patients what they value rather than trying to infer it. Age, sex, education or comorbidity are poor substitutes for an individual’s priorities and can introduce stereotypes into a model. A decision-support tool should allow patients to express how much they value treatment-free time, route of administration, monitoring intensity, avoidance of particular toxicities and other burdens [36,37,38,39]. A practical interface should first present clinically calibrated benefit and harm estimates under each eligible regimen on separate comparable scales and at the same time horizon; clinicians and patients can then filter options by unacceptable characteristics (e.g., avoid continuous therapy, avoid intravenous visits, or require a feasible ramp-up schedule). These filters are not demographic proxies and must not alter estimated causal risks. Preferences should be directly re-elicited at treatment initiation, new toxicity, progression, major functional or life changes, or subsequent sequencing decisions, with the date and source of each stated preference recorded. When a preferred option is clinically contraindicated or outside the data support, the interface explains why and invites shared reassessment rather than silently re-ranking an unsafe choice [14,36,37,38,39].

## 8. What AI Can Already Do in CLL?

AI is already being applied in CLL to several clinical and research tasks, including diagnostic and flow-cytometric classification, prediction of treatment need, prognostic modeling, MRD assessment, and analysis of high-dimensional molecular data. These applications demonstrate the potential of AI in CLL, but they address different questions from comparative treatment selection for an individual patient [9]. The expanded map in Table 4 distinguishes the task actually demonstrated (laboratory diagnosis, MRD measurement, early risk, time to treatment, survival, observed or ex vivo drug response) from the untested target of causal regimen comparison. It also includes studies omitted from the original illustrative table, including an operationally monitored CLL MRD system and an ex vivo drug-response study [40,41,42,43,44,45,46,47,48,49].

CLL-TIM is one of the strongest examples of clinically oriented ML in CLL. Using nationwide Danish data from 4149 newly diagnosed patients, the model applied an ensemble approach to identify patients at high risk of severe infection and/or treatment within two years [5]. Its handling of missing data, individualized risk factors, uncertainty estimates, and external validation provides useful methodological lessons for future decision-support models.

Meiseles and colleagues adopted a deliberately pragmatic approach, developing an explainable model to predict treatment need within two years using demographic and routine laboratory data from a single-center cohort of 109 patients. The model achieved an AUC of approximately 0.77 without genetic, fluorescence in situ hybridization (FISH), or flow-cytometric variables and used SHapley Additive exPlanations (SHAP) to provide individual-level explanations [6]. The study also illustrates an important limitation of this type of prediction: identifying which patients are likely to require treatment does not determine which treatment they should receive.

AI-assisted flow cytometry illustrates a different application. Human-in-the-loop deep neural networks have achieved high concordance with expert MRD assessment while reducing laboratory workload [7]. A more recent 2026 pipeline further demonstrated substantial reductions in manual analysis time while preserving low-level MRD detection [8]. These approaches may improve the efficiency and scalability of MRD assessment, but they remain measurement tools rather than treatment-selection models.

Transcriptomic machine-learning models have also been used to predict time to treatment with high performance in selected datasets [50]. Such high-dimensional approaches may eventually provide biological information beyond conventional clinical markers, although their transportability, cost, reproducibility, and incremental clinical value remain important considerations.

Table 4 provides a focused evidence map of 55 original CLL-relevant AI/ML records identified by screening the supplied PubMed search export. Study names and PubMed identifiers are shown for direct source retrieval. The selected evidence spans distinct tasks and must not be interpreted as 55 independently validated comparative treatment-selection algorithms.

**Table 4:** Focused evidence map of 55 original CLL-relevant AI/ML publications identified in the PubMed screening and their gaps for treatment selection.

Study [Reference]	Clinical/Analytical Task	Evidence Available in Exported Record	Remaining Gap for Individualized Selection
Simonson PD et al. (2022) [51] PMID 34724537	Diagnostic classification/workflow	Validation or hold-out assessment described in abstract	Diagnostic or methodological task, not comparative drug selection
Kendell I et al. (2026) [52] PMID 42443393	Drug-response or therapy optimization	Abstract performance/validation details not available in export	Exploratory/ex vivo or modeled response; clinical causal validation required
Argelaguet R et al. (2018) [53] PMID 29925568	Molecular stratification/high-dimensional data	Multi-omics integration in 200 CLL samples; ex vivo response data	Stratification does not identify treatment-specific benefit
Bigorra L et al. (2019) [54] PMID 29790152	Diagnostic classification/workflow	Abstract performance/validation details not available in export	Diagnostic or methodological task, not comparative drug selection
Parviz M et al. (2024) [30] PMID 38179708	Toxicity or complication prediction	Treatment-aware machine learning for AF risk at treatment initiation	Risk prediction is not a causal comparison between eligible regimens
Mauvieux L et al. (2025) [55] PMID 41236721	Diagnostic classification/workflow	Abstract performance/validation details not available in export	Diagnostic or methodological task, not comparative drug selection
Chen P et al. (2022) [56] PMID 35626003	Prognosis or outcome prediction	Abstract performance/validation details not available in export	Associative/prognostic prediction, not treatment-specific CATE
Meiseles A et al. (2022) [6] PMID 35405402	Prediction of treatment need	109 patients; internally assessed AUC approximately 0.77	Predicts whether treatment is needed, not which therapy is preferable
Dinalankara W et al. (2026) [57] PMID 41700529	MRD measurement/flow laboratory	Abstract performance/validation details not available in export	Analytical measurement, not treatment-effect estimation
Coombes CE et al. (2020) [58] PMID 32483590	Prognosis or outcome prediction	Original application; validation/calibration not established from the available abstract	Associative/prognostic prediction, not treatment-specific CATE
Agius R et al. (2020) [5] PMID 31953409	Toxicity or complication prediction	4149 newly diagnosed CLL patients; infection/need prediction; external testing	Risk prediction is not a causal comparison between eligible regimens
Li MX et al. (2025) [48] PMID 41476951	Drug-response or therapy optimization	133 primary CLL samples; high-content imaging and ex vivo response clusters	Exploratory/ex vivo or modeled response; clinical causal validation required
Ng DP et al. (2021) [59] PMID 33210119	Diagnostic classification/workflow	Original application; validation/calibration not established from the available abstract	Diagnostic or methodological task, not comparative drug selection
Zhao M et al. (2020) [40] PMID 32519455	Diagnostic classification/workflow	20,622 mixed B-cell neoplasm flow-cytometry cases; weighted F1 0.94	Diagnostic or methodological task, not comparative drug selection
Mosquera Orgueira A et al. (2019) [50] PMID 30828568	Prediction of treatment need	Abstract performance/validation details not available in export	Forecasts when treatment is needed, not which regimen is optimal
Zhu Y et al. (2022) [60] PMID 36467501	Diagnostic classification/workflow	Validation or hold-out assessment described in abstract	Diagnostic or methodological task, not comparative drug selection
deAndrés-Galiana EJ et al. (2016) [61] PMID 26956213	Prognosis or outcome prediction	Original application; validation/calibration not established from the available abstract	Associative/prognostic prediction, not treatment-specific CATE
Carreras J et al. (2022) [62] PMID 36358737	Prognosis or outcome prediction	Abstract performance/validation details not available in export	Associative/prognostic prediction, not treatment-specific CATE
Turcsanyi P et al. (2019) [63] PMID 30852300	Prognostic risk stratification	Original application; validation/calibration not established from the available abstract	Risk stratification does not estimate comparative regimen effects
Parviz M et al. (2022) [64] PMID 35468622	Prognosis or outcome prediction	Abstract performance/validation details not available in export	Associative/prognostic prediction, not treatment-specific CATE
Ladyzynski P et al. (2022) [65] PMID 35110619	Health-status and treatment-effect modeling	Abstract performance/validation details not available in export	Dynamic outcome modeling; causal regimen comparison not established
Taus P et al. (2021) [66] PMID 34262590	Molecular subgroup/pathway discovery	Abstract performance/validation details not available in export	Molecular subtyping does not estimate regimen-specific clinical benefit
El Hussein S et al. (2022) [67] PMID 35132162	Histopathology: proliferation-center mapping	Abstract performance/validation details not available in export	Diagnostic pathology distinction, not treatment-effect estimation
Tang G et al. (2021) [68] PMID 34350848	Digital microscopy: abnormal lymphocytes	Abstract performance/validation details not available in export	Morphologic detection, not comparative drug selection
Haider RZ et al. (2022) [69] PMID 35054304	CBC-based leukemia differentiation	Abstract performance/validation details not available in export	Diagnostic classification, not comparative drug selection
Aoki J et al. (2025) [45] PMID 40554740	Early lymphocytosis risk prediction	1,090,707 laboratory records; held-out test set; AUC 0.92	Predicts abnormal lymphocytosis, not a therapeutic choice
Salama ME et al. (2022) [7] PMID 35626140	MRD measurement/flow laboratory	236 flow-cytometry samples; human-in-the-loop MRD workflow	Analytical measurement, not treatment-effect estimation
Sathitakorn O et al. (2025) [70] PMID 41059584	Diagnostic classification/workflow	Original application; validation/calibration not established from the available abstract	Diagnostic or methodological task, not comparative drug selection
Hoffmann J et al. (2023) [41] PMID 36826109	Prognosis or outcome prediction	157 patients; AUC 0.95 versus 0.78 for CLL-IPI	Associative/prognostic prediction, not treatment-specific CATE
Padmanabhan R et al. (2023) [71] PMID 37387017	Diagnostic classification/workflow	Abstract performance/validation details not available in export	Diagnostic or methodological task, not comparative drug selection
Christopoulos G et al. (2024) [72] PMID 38774001	Toxicity or complication prediction	AI-derived electrocardiogram (ECG) score tested for AF prediction in CLL	Risk prediction is not a causal comparison between eligible regimens
Mouazer A et al. (2025) [46] PMID 40200453	Prognosis or outcome prediction	Abstract performance/validation details not available in export	Associative/prognostic prediction, not treatment-specific CATE
Yin Y et al. (2025) [73] PMID 40085050	Drug-response or therapy optimization	Validation or hold-out assessment described in abstract	Exploratory/ex vivo or modeled response; clinical causal validation required
Nguyen PC et al. (2023) [43] PMID 37052611	Diagnostic classification/workflow	456 MRD flow-cytometry samples; agreement with expert analysis	Diagnostic or methodological task, not comparative drug selection
Bazinet A et al. (2024) [42] PMID 36824056	MRD measurement/flow laboratory	113 MRD samples; separate training and validation samples	Analytical measurement, not treatment-effect estimation
Chen D et al. (2019) [74] PMID 31112417	Prediction of treatment need	Original application; validation/calibration not established from the available abstract	Forecasts when treatment is needed, not which regimen is optimal
Chiu A et al. (2026) [8] PMID 42192141	MRD detection/flow cytometry	227 MRD files; R^2^ 0.98; manual analysis time reduced by approximately 90%	MRD measurement and workflow, not clinical treatment-effect estimation
Vanderkerken M et al. (2026) [75] PMID 41849725	Real-world data extraction and cohort description	Original application; validation/calibration not established from the available abstract	Observational cohort characterization, not regimen-effect estimation
Vergnolle I et al. (2025) [76] PMID 37539849	Flow-cytometry diagnostic decision tree	Original application; validation/calibration not established from the available abstract	Diagnostic subtype classification, not treatment selection
Lin J et al. (2025) [77] PMID 39627967	Diagnostic classification/workflow	Abstract performance/validation details not available in export	Diagnostic or methodological task, not comparative drug selection
Arakelyan A et al. (2025) [78] PMID 40149301	Molecular stratification/high-dimensional data	Abstract performance/validation details not available in export	Stratification does not identify treatment-specific benefit
Morabito F et al. (2023) [79] PMID 37719021	Molecular stratification/high-dimensional data	Abstract performance/validation details not available in export	Stratification does not identify treatment-specific benefit
Wang Y et al. (2023) [80] PMID 37244059	Peripheral-blood morphology analysis	Performance metric reported in abstract; direct comparability is limited	Morphologic monitoring, not comparative drug selection
Xiao X et al. (2021) [81] PMID 34139331	Prediction of treatment need	Abstract performance/validation details not available in export	Forecasts when treatment is needed, not which regimen is optimal
Moysiadis T et al. (2023) [82] PMID 37664638	Time-to-first-treatment prediction	Abstract performance/validation details not available in export	Forecasts treatment timing, not comparative regimen benefit
Lorenzo MCC et al. (2026) [83] PMID 41560465	Single-cell morphometric classification	Abstract performance/validation details not available in export	Morphometric characterization, not comparative drug selection
Papangelou C et al. (2026) [49] PMID 42174951	Prognosis or outcome prediction	Development 4472 and real-world 5616; model transfer and uncertainty	Associative/prognostic prediction, not treatment-specific CATE
Esposito F et al. (2025) [84] PMID 40428274	Prognosis or outcome prediction	Validation or hold-out assessment described in abstract	Associative/prognostic prediction, not treatment-specific CATE
Seheult JN et al. (2025) [47] PMID 40427185	MRD measurement/flow laboratory	240 validation samples; 874 post-implementation QC samples; operational monitoring	Analytical measurement, not treatment-effect estimation
Thrun MC et al. (2026) [85] PMID 42441729	Diagnostic classification/workflow	Validation or hold-out assessment described in abstract	Diagnostic or methodological task, not comparative drug selection
Parviz M et al. (2026) [86] PMID 41711490	Toxicity or complication prediction	Domain-adaptation ML for infection prediction in treated CLL	Risk prediction is not a causal comparison between eligible regimens
Tian Z et al. (2026) [87] PMID 41733051	Immunohistochemical differential diagnosis	Abstract performance/validation details not available in export	Mantle cell lymphoma versus CLL/SLL diagnosis; not therapy selection
Tadmor T et al. (2025) [88] PMID 40071509	Toxicity or complication prediction	ML-derived CLLAF AF risk score at BTKi treatment initiation	Risk prediction is not a causal comparison between eligible regimens
Wang Z et al. (2026) [89] PMID 42184194	Drug-response or therapy optimization	Simulation/digital twin for adaptive venetoclax-resistance policy	Exploratory/ex vivo or modeled response; clinical causal validation required
Zhang X et al. (2026) [90] PMID 41865503	In-hospital mortality prediction	Interpretable inpatient mortality score with temporal validation	Hospital mortality risk prediction, not comparative treatment effects

The mapped literature spans highly heterogeneous targets, from low-level MRD measurement and flow-cytometric classification to infection, atrial fibrillation, survival and treatment-need prediction. Large diagnostic cohorts and high discrimination do not establish comparative regimen utility; smaller studies additionally raise concerns about event counts, model complexity and optimism. Evaluation of raw cell-level or repeated-sample data requires patient-level partitioning to avoid leakage, while historical treatment-specific prediction requires correction for confounding by indication. Methodological reporting was inconsistent across accessible abstracts, and absent details are not taken as evidence that a method was never used [9,12,15]; notably, treatment-aware AF risk [30], post-treatment infection prediction [86], and CLL-directed adaptive therapy simulation [89] remain distinct from validated causal regimen choice.

## 9. From Outcome Prediction to Individualized Treatment Effects: A Causal Specification for CLL

Suppose a model estimates a high probability of cardiovascular toxicity for a patient receiving a BTK inhibitor (BTKi). This is clinically useful information, but it does not establish whether a venetoclax-based strategy would lead to a better overall outcome for the same patient. Treatment selection is inherently counterfactual: the relevant question is what outcome would occur under treatment A compared with treatment B, even though only one of these potential outcomes can be observed for any individual patient [11]. Define A as a clinically feasible regimen (e.g., A = 1 continuous BTK inhibition and A = 0 fixed-duration venetoclax-based therapy), X as pre-treatment covariates, and Y(a,t) as a potential outcome by a prespecified horizon t had regimen a been initiated. Only Y(A,t) is observed for a given patient. Individual potential-outcome pairs are fundamentally unobservable; the estimable population target is the conditional average treatment effect (CATE), expressed as CATE(x,t) = E[Y(1,t) − Y(0,t) | X = x]. For binary adverse events, the contrast is an absolute risk difference; for survival, one can instead use a difference in t-year survival probabilities or restricted mean survival time. This quantity is not an accurately measurable benefit for a specific patient: it is an estimated mean contrast among patients sharing a covariate profile [10,11,91].

This distinction separates conventional outcome prediction from individualized treatment-effect estimation. Standard supervised-learning models can predict outcomes among patients who received a particular treatment, but treatment assignment in routine clinical practice is not random. Clinicians may avoid a BTK inhibitor in a patient with recurrent atrial fibrillation or hesitate to initiate venetoclax in a patient with renal impairment and bulky disease. Without appropriate causal methods, an algorithm may therefore learn historical treatment-selection patterns and the confounding embedded within them rather than the causal effect of treatment [10,11,91]. Identification from observational data requires consistency and adequately defined treatment strategies, exchangeability conditional on measured confounders, treatment overlap/positivity, a common time zero, and correct measurement of exposure, outcomes and censoring. In CLL, treatment choice reflects TP53, previous therapy, arrhythmias, anticoagulation, renal function, tumor burden, infection vulnerability and prescriber or center preferences. Conditioning only on an aggregated risk score is not sufficient if clinically decisive confounders remain unrecorded. Prescriber/center effects should be represented transparently rather than interpreted as intrinsic patient-level treatment effects [10,11,91].

For an explicit target-trial emulation, define (1) an indication-based eligibility date; (2) comparator regimens available in the same calendar era; (3) treatment assignment at time zero with a clinically justified grace period; (4) baseline covariates measured before assignment; (5) endpoints and common follow-up horizon (e.g., three-year PFS, grade ≥3 adverse events, discontinuation, hospitalization); and (6) an intention-to-treat-like initiation contrast or a separate per-protocol sustained-strategy contrast. Confounding by indication is addressed by estimating a propensity score e(X) = Pr(A = 1|X) and using inverse-probability treatment weights A/e(X) + (1 − A)/(1 − e(X)), or stabilized counterparts, followed by covariate-balance and extreme-weight diagnostics. Matching, outcome regression, g-computation and doubly robust methods offer alternative sensitivity analyses. Weighting reduces measured confounding under assumptions; it does not remove unmeasured prescriber bias or repair absent treatment overlap [10,11,91].

Time-varying confounding is especially relevant to CLL sequencing: renal function, cytopenias, atrial fibrillation, infection, MRD, intolerance and disease burden evolve and can both influence later treatment changes and be consequences of earlier therapy. Ordinary adjustment for such post-baseline variables may block part of treatment effects or create collider bias. When the estimand concerns dynamic treatment sequences, strategies must be specified at repeated decision times and analyzed using sequential target-trial emulation, marginal structural models with treatment/censoring weights, or longitudinal g-methods, subject to sequential exchangeability and positivity. Switching from BTKi to a BCL2 inhibitor (BCL2i) (or vice versa) is not exchangeable with fixed-duration treatment simply because both appear in a record; immortal-time and treatment-line misclassification must be prevented. Treatment-specific CATE should be estimated within a well-supported decision context, checked for robustness, and abstain when support is inadequate [10,11,91].

Validation must include causal plausibility, predeclared effect modifiers, internal bootstrap optimism correction, site-held-out and temporal cohorts, overlap diagnostics, calibration of treatment-specific outcome risks and, when defensible, calibration of treatment-benefit estimates. Randomized evidence should anchor treatment contrasts whenever available. Heterogeneity estimates from flexible causal forests, meta-learners or doubly robust learners require shrinkage, uncertainty intervals and tests against simpler prespecified subgroup models; a favorable SHAP attribution is neither a causal explanation nor an unbiased individual treatment effect [11,12,15,91].

## 10. A Patient-Specific Toxicity and Benefit Profile

For individualized treatment selection, a single generic toxicity score is unlikely to provide sufficient information. A more clinically useful approach would be a patient-specific profile encompassing cardiovascular risk, bleeding risk, renal function and TLS risk, hematologic and infectious vulnerability, frailty or functional reserve, and relevant drug interactions. Treatment burden and logistical feasibility should also be represented explicitly, as they may substantially influence treatment acceptability despite not being conventional toxicity endpoints [1,2,16,26,33,34].

The definition of the outcome is equally important. A transient and manageable grade 2 adverse event is clinically different from an event resulting in hospitalization, permanent treatment discontinuation, irreversible morbidity, or loss of independence. Models should therefore characterize not only the occurrence of toxicity, but also its severity, duration, consequences for treatment delivery, risk of discontinuation, and patient-reported impact.

For each clinically reasonable treatment option, model output should present several dimensions separately before any attempt is made to summarize them. These may include expected disease control, treatment-specific toxicity, probability of treatment discontinuation, treatment burden, and uncertainty. Collapsing these domains into a single score may obscure the relative importance assigned to different outcomes, which should remain part of shared decision-making between the clinician and patient [14,36,37,38,39].

The clinical relevance of such multidimensional assessment can be illustrated by patients with similar leukemia-related characteristics but markedly different vulnerability profiles. One patient may be 72 years old, have unmutated IGHV and no TP53 disruption, but a history of atrial fibrillation requiring chronic anticoagulation. A second patient with the same age and molecular profile may have preserved cardiovascular function but chronic kidney disease and bulky lymphadenopathy. A third may have recurrent respiratory infections, hypogammaglobulinemia, and chronic lung disease. Although their disease biology may be similar, the clinical consequences of available treatment options may differ substantially.

## 11. Explainability, Calibration and Uncertainty

A clinically useful decision-support system should not provide a treatment recommendation without also showing the alternatives considered and the factors contributing to differences between them. If previous atrial fibrillation, anticoagulation, and uncontrolled hypertension account for much of the predicted risk associated with one strategy, this should be apparent to the clinician. The same principle applies when renal impairment and tumor burden contribute substantially to initiation-related risk with another treatment [12,14].

Explainability, however, does not establish model validity. SHAP values and related attribution methods describe how input variables contribute to a prediction, but they do not demonstrate causality, correct poor calibration, or compensate for bias in study design. A model may therefore be interpretable while remaining clinically inaccurate or systematically biased [11,12,14].

Calibration is particularly important when absolute risk estimates are used in shared decision-making. A model may rank patients correctly while substantially misestimating their absolute risk. For example, systematically estimating a true 10% toxicity risk as 30% could materially influence treatment discussions. Reporting should therefore follow contemporary standards such as Transparent Reporting of a multivariable prediction model for Individual Prognosis Or Diagnosis–Artificial Intelligence (TRIPOD + AI), with transparent description of data provenance, preprocessing, handling of missing data, model development, calibration, discrimination, validation, and fairness [12,14].

Uncertainty should also be communicated at the individual-patient level. Predictions for patients who differ substantially from the development population should not be presented with the same apparent precision as estimates for well-represented patients. Decision-support systems should therefore be able to indicate low-confidence predictions and, where appropriate, refrain from favoring one treatment option when data are sparse, treatment overlap is inadequate, or the available evidence does not reliably distinguish between alternatives [14].

## 12. Building a Clinically Useful Model

Model development should begin with a clearly defined clinical decision rather than with the choice of a particular algorithm. The disease-related layer should include TP53 mutation/del(17p), IGHV mutational status, disease burden, cytopenias, treatment history, and, where relevant, additional genomic information. An equally important patient-related layer should capture frailty, functional status, renal function, cardiovascular disease, bleeding and infection history, immune status, comorbidity, and concomitant medications [1,2,16].

The treatment layer should represent the specific drug or regimen rather than only its pharmacologic class, together with treatment duration, monitoring requirements, drug–drug interactions, previous intolerance, and resistance. A model that groups ibrutinib, acalabrutinib, and zanubrutinib simply as “BTK inhibitors” may obscure clinically relevant safety differences demonstrated in randomized and real-world comparisons [26,27,28,29].

The outcome layer should estimate treatment-specific efficacy together with clinically meaningful harms. Patient preferences should be represented separately so that individuals can indicate the relative importance they assign to treatment duration, route of administration, treatment-free time, monitoring requirements, avoidance of specific toxicities, and other treatment burdens. The user interface should then allow reasonable treatment alternatives to be compared directly, with uncertainty estimates and explanations presented alongside the predicted outcomes [14,36,37,38,39].

Missing data require explicit consideration because missingness in real-world CLL datasets may reflect patterns of clinical care. Frailty, for example, may be recorded more frequently in older patients; molecular testing may depend on treatment era or center; and infections managed outside hematology may be absent from structured records. Statistical imputation can address missing values, but it cannot fully correct the bias introduced when clinically important information is systematically unavailable [12,92].

Temporal relevance is equally important. Models developed predominantly from chemoimmunotherapy-era or early ibrutinib-era data may not generalize to second-generation BTK inhibitors, BTK inhibitor–BCL2 inhibitor combinations, or newer sequencing strategies. Temporal validation should therefore be incorporated into model evaluation, and strategies for model updating should be defined prospectively [14,93].

Table 5 summarizes minimum requirements for the development and evaluation of clinically trustworthy AI-assisted treatment-selection tools in CLL [12,13,14,92,93].

Before implementation in routine care, a decision-support system should undergo prospective evaluation of workflow integration, human factors, safety, and unintended consequences, as emphasized by Developmental and Exploratory Clinical Investigations of DEcision-support systems driven by Artificial Intelligence (DECIDE-AI) [13]. If the system is subsequently evaluated in an interventional trial, AI-specific reporting standards such as Standard Protocol Items: Recommendations for Interventional Trials–Artificial Intelligence (SPIRIT-AI) and Consolidated Standards of Reporting Trials–Artificial Intelligence (CONSORT-AI) become relevant [14,94,95,96].

Comparison with existing frameworks clarifies where the present CLL proposal extends rather than duplicates prior work. Precision-oncology tumor boards prioritize actionable variants and levels of evidence; clinical-pathway tools improve order appropriateness and guideline adherence; multi-criteria approaches explicitly structure values, but do not thereby estimate CATE. Reporting and governance instruments assess distinct stages of model development and deployment rather than supply a disease-specific causal treatment-ranking algorithm [12,13,14,15,92,94,95,97,98,99,100]. These comparisons and the additional requirements of the proposed CLL-specific framework are summarized in Table 6.

## 13. Proposed Architecture for AI-Assisted, Clinician-Supervised Treatment Selection

The proposed framework integrates six components: disease characteristics, patient vulnerability, treatment context, treatment-specific efficacy and toxicity estimates, model calibration and uncertainty, and patient preferences. These components should not function as independent prediction modules, but as parts of a single decision-support pathway that compares clinically reasonable treatment options for an individual patient [14]. The novelty claimed here is compositional and operational: (a) treatment-eligibility gating, (b) a target-trial-defined comparator and common horizon, (c) model validation and explicit abstention, and (d) preference filtering after estimation. These components form a conceptual research specification. No authors have trained the integrated model, estimated its discrimination or treatment effects, or demonstrated that it improves clinical outcomes.

Disease-related information includes TP53 mutation/del(17p), IGHV mutational status, disease burden, cytopenias, treatment history, and additional genomic features where available. Patient-level information includes frailty, functional status, renal function, cardiovascular disease, bleeding risk, infection history, immune status, comorbidity, and concomitant medications. Treatment context should capture the specific regimen, treatment duration, monitoring requirements, drug interactions, previous intolerance, and resistance [1,2,16,26,33,34].

Treatment-specific models can then estimate expected efficacy, clinically meaningful toxicity, treatment discontinuation, and treatment burden for each relevant option. These estimates should be presented together with calibration, uncertainty, and interpretable explanations. Patient preferences are incorporated as a separate input so that treatment duration, route of administration, monitoring intensity, treatment-free time, and avoidance of specific toxicities can be considered explicitly [11,12,36,37,38,39].

The final output should be a side-by-side comparison of reasonable treatment strategies rather than a single unqualified recommendation. The purpose of the system is to support shared decision-making by making differences in expected benefit, harm, burden, and uncertainty transparent to both clinician and patient [2,14]. The proposed pipeline displays treatment-specific risks and benefits first; it never incorporates patient preferences as latent predictors of medical response or as unvalidated subjective weights inside the causal model. At a separate post hoc, clinician–patient dialogue stage, participants can mark unacceptable therapies or logistical burdens and consider remaining clinically eligible choices. If multiple remain, the interface supports deliberation rather than forcing a unique rank. Preference dates and reassessment triggers are stored so that changes in values do not contaminate treatment-risk calibration [36,37,38,39].

Distinct output panels should show, for every eligible regimen at the same horizon, estimated progression-free survival, serious toxicity risk, permanent discontinuation probability, treatment visits/monitoring, calibration state, intervals of uncertainty and population overlap. The output should explicitly display “insufficient comparable evidence” and abstain if any treatment-specific probability or CATE falls outside validated support. Calibration of clinical risk estimates occurs before preference filtering; post hoc filtering changes discussion priorities, not predicted event probabilities.

Figure 2 retains the original illustrated two-panel conceptual framework. Its patient-preference component should be interpreted as a post-prediction shared-decision-making step: patient priorities do not alter clinical risk calibration. The figure represents a proposed architecture rather than an empirically validated algorithm.

### Illustrative Clinical Vignette: Same Disease, Different Feasibility

Consider a hypothetical 72-year-old patient with previously untreated symptomatic CLL, unmutated IGHV, no TP53 disruption, atrial fibrillation requiring anticoagulation, chronic kidney disease (estimated glomerular filtration rate [eGFR] 45 mL/min/1.73 m^2^), and bulky lymphadenopathy. The first step is not algorithmic ranking: a hematologist confirms the indication for therapy and identifies feasible, guideline-supported regimens, taking into account local availability and contraindications. The two vulnerabilities create opposing issues: a BTKi-containing strategy may raise cardiovascular and bleeding concerns, whereas venetoclax initiation may require enhanced TLS monitoring and feasible rapid access to laboratory assessment [1,2,26,36]. This combination makes simplistic “BTKi for patient A; venetoclax for patient B” rules unsafe.

The proposed decision-support interface would show hypothetical, non-numeric treatment-specific outputs under each eligible regimen: 3-year PFS, risk of hospitalization-requiring cardiovascular complications, TLS-related initiation burden, infection, treatment discontinuation, and associated evidence/uncertainty. Because no validated integrated model currently supplies these estimates, the authors deliberately do not invent numeric individualized probabilities or a preferred treatment. The clinician then asks whether the patient accepts a prolonged oral regimen, wishes to avoid infusion visits, can attend TLS surveillance, and how their preferences might change if employment, health, or caregiving circumstances change. Preferences filter the medically supported options after estimation, rather than change the model coefficients. If external validation or treatment-overlap criteria fail, the system explicitly abstains. Reassessment at toxicity, progression, or a new treatment line restarts the eligibility and causal-comparator definition.

## 14. Linking Diagnostic and Prognostic Information to Treatment Decisions

Diagnostic and prognostic information becomes clinically actionable only when interpreted in relation to a specific treatment decision. Molecular findings, laboratory characteristics, disease burden, and MRD can refine risk assessment in CLL, but their relevance to treatment selection depends on the therapy being considered and on the vulnerability of the individual patient. Within the proposed framework, biomarkers therefore contribute to treatment comparison rather than functioning as isolated determinants of treatment choice [1,16,101].

The same principle applies to model interpretation. Clinicians should be able to identify which disease-related, prognostic, and patient-level characteristics contribute meaningfully to differences in expected outcomes across treatment options. Equally important, the system should indicate when available data are insufficient to support a reliable, individualized comparison [12,14].

The next step beyond increasingly accurate classification and prognosis is therefore to connect disease-related information with patient context and treatment-specific outcomes. The aim is not simply to predict what is likely to happen, but to make the consequences of alternative treatment strategies more explicit and clinically interpretable [2,16].

## 15. Implementation Barriers, Methodological Appraisal and Clinical Utility

Important barriers remain before AI-assisted treatment selection can be evaluated for routine CLL care. Most published models continue to focus on diagnosis, prognosis, or treatment need, and many available cohorts are too small to estimate credible differences in treatment effects across clinically relevant patient subgroups. External and temporal validation remain inconsistent, while toxicity and patient-reported outcomes are often captured less completely than conventional efficacy endpoints [9,14,96].

Data fragmentation may represent an even greater challenge than model complexity. Cardiovascular events may be managed outside hematology, infections treated at other institutions, and functional decline may never be recorded in structured datasets. Drug exposure, dose reductions, treatment interruptions, and reasons for discontinuation may also be difficult to reconstruct reliably. Linking hematology, pharmacy, hospitalization, primary-care, and other relevant data sources may therefore provide greater clinical value than applying increasingly complex algorithms to incomplete datasets [92,93,94,95,96].

Fairness also requires explicit evaluation. If access to molecular testing, specialist care, or particular therapies differs between centers or patient groups, an algorithm may reproduce disparities already present in the healthcare system. Model performance should therefore be examined across clinically relevant subgroups, including age, sex, comorbidity, and treatment center, rather than assuming that acceptable aggregate performance implies comparable performance for all patients [14,92].

Ultimately, prospective evaluation must establish whether decision support improves clinical decision-making rather than prediction alone. Relevant outcomes may include concordance with evidence-based treatment options, avoidable severe toxicity, treatment discontinuation, decisional conflict, quality of life, and clinician workload. Improvements in discrimination metrics such as AUC are insufficient on their own to demonstrate clinical benefit [13,14,96]. Concrete, independently adjudicated endpoints should be prespecified before a prospective study. Treatment concordance can be defined as the proportion of initiation decisions judged appropriate by a blinded expert panel against contemporaneous CLL guidelines and documented contraindications; it is not agreement with the model itself. Avoidable grade ≥3 toxicity can be assessed by independent adverse-event adjudication using a prespecified preventability rubric and the number of events per 100 treated patients over 12 months. Permanent discontinuation should require documented reasons, competing-risk handling, and a fixed follow-up horizon. Patient-reported decisional conflict can be captured with the validated Decisional Conflict Scale at the decision visit, alongside quality-of-life instruments at baseline and defined follow-ups. Workflow burden includes time to decision, clinician interaction time, overridden suggestions and delay to treatment. A prospective cluster-randomized or patient-randomized trial can compare standard multidisciplinary shared decision-making versus the same process supported by the audited interface [13,94,95,100].

Predictive discrimination (AUC/concordance index [C-index]) is necessary in some tasks but insufficient for decision quality. Before any clinical deployment, each regimen-specific estimate requires calibration plots, calibration-in-the-large (intercept), calibration slope, observed-to-expected ratios at the relevant horizon, Brier score, confidence intervals, and reporting by site, calendar era and relevant clinical subgroups. Decision-curve net benefit must be plotted over clinically defensible action thresholds derived from the real decision (e.g., additional cardiovascular surveillance or monitoring escalation), not arbitrary thresholds for choosing one regimen. For multi-attribute treatment selection, a transparent utility analysis can quantify the decision consequences of outcome-specific errors and patient-stated acceptability after valid medical prediction. Net-benefit difference versus treat-all/treat-none or usual-care comparators where those comparators are meaningful can be evaluated; for multi-arm regimen choice, present policy-value and regret analyses only if outcomes and preferences are identified and validated. The prospective trial should report safety, equity, workflow harms, calibration drift, overrides, and the proportion of patients for whom the model abstains [12,13,14,15,100].

A formal PROBAST + AI assessment of every historical paper is not claimed without source-level primary-report re-extraction. The descriptive appraisal nevertheless identifies mandatory checks for each candidate model: patient-level rather than aliquot-level splitting; number of outcome events and effective model complexity rather than a universal 10-events-per-variable rule; complete data provenance; feature timing before the index date; leakage through repeated samples, future MRD or post-treatment measurements; missing-data mechanism and multiple imputation within resampling; optimism correction by nested resampling/bootstrap; independent site and temporal validation; model calibration and net clinical benefit. An independent external cohort must share the clinical decision, eligibility and accessible treatment options. These checks are summarized in Table 7 and build directly on TRIPOD + AI, PROBAST + AI and prospective AI guidance [12,13,14,15,94,95].

## 16. Future Directions: From Precision Treatment to Precision Care

Future CLL decision-support models will likely need to be dynamic rather than limited to a single baseline assessment. Renal function, cardiovascular status, infectious vulnerability, frailty, and other patient characteristics may change over time, while the disease itself evolves under treatment pressure. Longitudinal models could therefore update treatment-specific estimates as new information becomes available rather than treating treatment initiation as the only clinically relevant decision point [14,93]. This roadmap requires re-estimation of causal contrasts when both prescribing and the available regimen set change; model maintenance cannot be reduced to intercept recalibration when novel therapies make historical treatment comparisons obsolete.

MRD may contribute to such a dynamic framework, particularly during and after time-limited therapy. FLAIR demonstrates the feasibility and clinical relevance of MRD-guided treatment duration in a randomized setting [20,21], while CLL14 and CLL17 illustrate the depth of response achievable with fixed-duration strategies [17,23]. The role of MRD in decision-support models remains treatment-context dependent, however, and should not be generalized across regimens without supporting evidence [101].

Multimodal models may eventually integrate routine clinical variables, longitudinal laboratory measurements, genomic information, flow cytometry, medication exposure, and patient-reported outcomes. Greater complexity, however, should not be considered an objective in itself. If a simpler model based on routinely available data provides comparable calibration, transportability, and clinical utility, its simplicity may improve implementation rather than represent a limitation [14,96].

Patient preferences should also be collected prospectively as clinical data in their own right. A future decision-support system could distinguish between the strategy associated with the greatest predicted disease-related benefit and the strategy that best aligns with an individual patient’s priorities after toxicity, treatment burden, and uncertainty are considered. This distinction is central to the transition from precision treatment to precision care [36,37,38,39].

## 17. Conclusions

CLL treatment has entered an era in which several highly effective therapeutic strategies may be clinically reasonable for the same patient. As a result, treatment selection has become increasingly dependent on factors beyond disease control alone [1,2,16].

Disease biology remains fundamental. TP53 disruption, IGHV mutational status, and other molecular features continue to inform prognosis and treatment selection, but they capture only part of the clinical decision. Cardiovascular disease, bleeding risk, renal function, infectious vulnerability, frailty, concomitant medications, and treatment burden may substantially influence the consequences of an otherwise effective regimen. Patient preferences represent an additional and distinct dimension that cannot be reliably inferred from clinical characteristics alone [1,2,16,36,37,38,39].

AI may help integrate these different sources of information, but prediction and treatment selection must remain conceptually distinct. Identifying patients who are likely to progress, require treatment, or experience toxicity does not determine which therapy offers the most appropriate benefit–risk balance for an individual patient. Comparative decision support will require treatment-specific estimates of efficacy and toxicity, appropriate causal methods, external and temporal validation, clinically meaningful endpoints, and explicit representation of uncertainty [10,11,41]. The study did not develop a new algorithm, evaluate an external dataset, or demonstrate superior treatment recommendations. Its original value is a falsifiable and operational CLL-specific specification with pre-selection eligibility safeguards, defined causal estimands, transparent uncertainty, and post hoc longitudinal preference elicitation.

Explainability is necessary but not sufficient for clinical trustworthiness. A model may be interpretable while remaining biased, poorly calibrated, or based on inappropriate causal assumptions. Its clinical utility must therefore be demonstrated through prospective evaluation in real-world decision-making [12,14].

The objective is not automated prescribing, but better-informed shared decision-making. A clinically useful system should help patients and clinicians compare reasonable treatment options in terms of expected disease control, toxicity, treatment burden, and uncertainty, while making the factors driving those estimates transparent. In this way, AI may contribute to the transition from precision treatment, focused primarily on disease characteristics, toward precision care, centered on the individual patient [2,14,36,37,38,39].

## Figures and Tables

**Figure 1:**
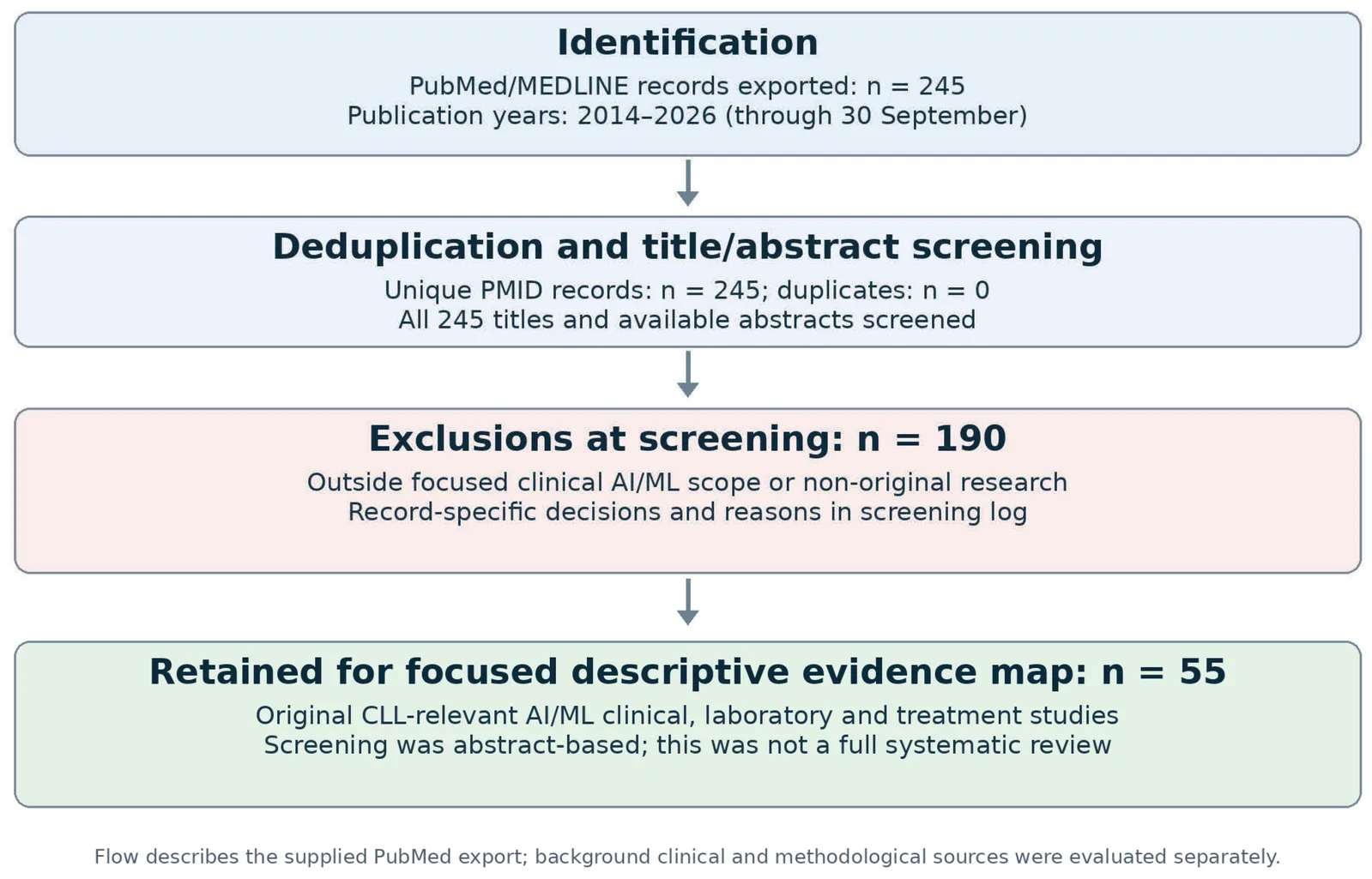
Identification and title/abstract screening of PubMed records for the focused evidence map. The database export included 245 unique records (0 duplicates). A total of 190 were excluded using the stated focused eligibility criteria; 55 original CLL-relevant AI/ML reports were retained for descriptive mapping. Clinical trials, guidelines and general methodological publications were synthesized separately. This diagram does not represent a full systematic review.

**Figure 2:**
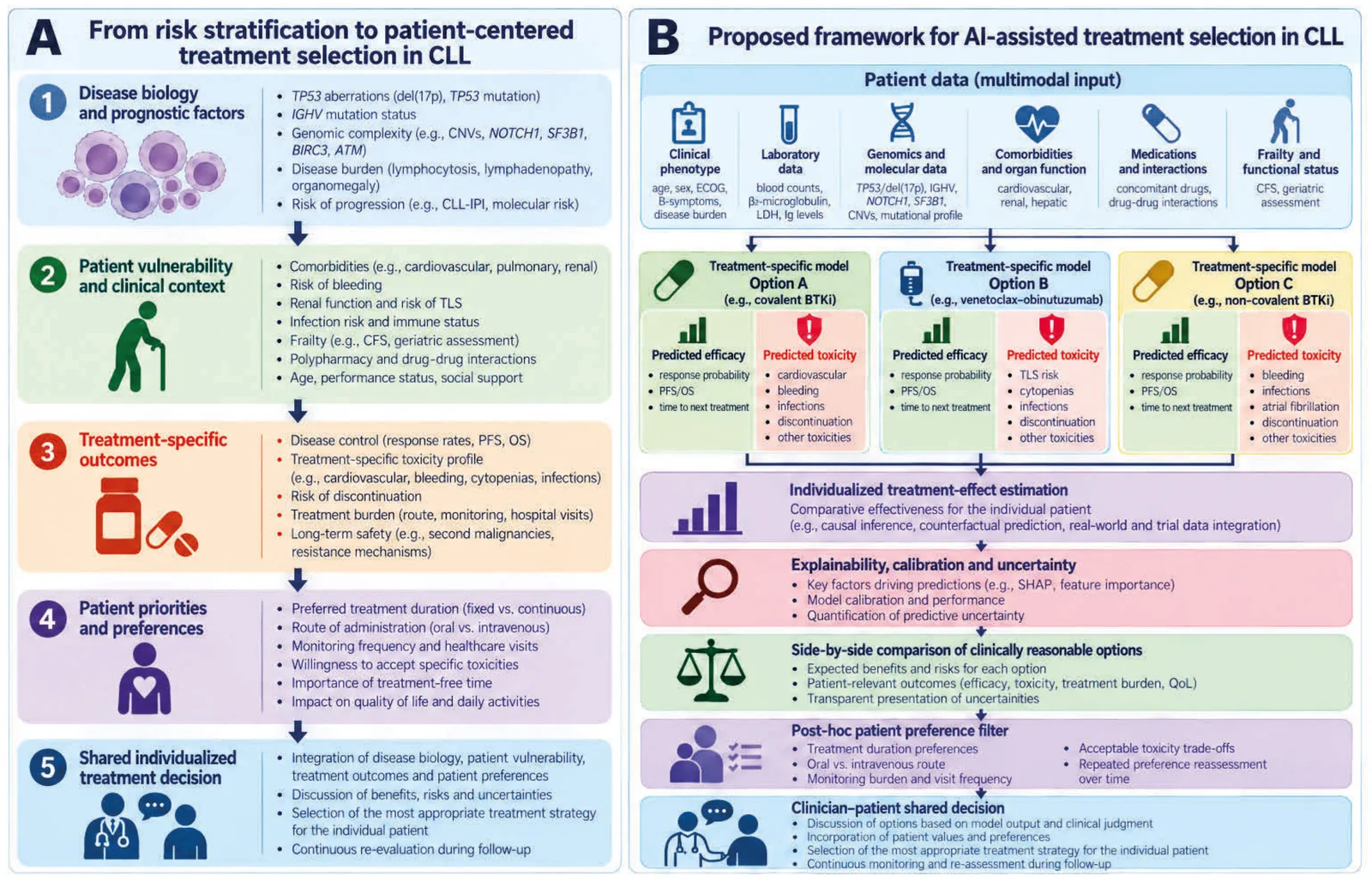
Original illustrated conceptual framework for AI-assisted treatment selection in chronic lymphocytic leukemia. (**A**) From conventional stratification toward patient-centered therapeutic choice. (**B**) Integration of patient, disease and treatment factors with treatment-specific outputs and shared decision-making. Patient preferences are elicited separately after clinical risk estimation.

**Table 1:** Variables relevant to individualized CLL treatment selection.

Factor	Established Role	Potential Relevance to Treatment Selection	Important Limitation
TP53 mutation/del(17p)	Major adverse biological risk factor	Essential before treatment selection and sequencing	Does not alone determine the optimal choice among all targeted options
IGHV mutational status	Established prognostic marker	Relevant to biological characterization and interpretation of treatment evidence	Its therapeutic role differs from the chemoimmunotherapy era
Complex karyotype/genomic complexity	Adverse risk feature in several settings	May refine high-risk characterization	Definitions and treatment-predictive value remain incompletely standardized
Disease burden	Reflects disease activity	Important for venetoclax tumor lysis syndrome (TLS) assessment and monitoring intensity	Treatment-specific rather than universal selector
MRD	Sensitive measure of response depth	Potentially useful for dynamic modeling and time-limited therapy	Clinical meaning depends on treatment context
Cardiovascular profile	Patient vulnerability domain	Important when BTK inhibition is considered	Risk differs between individual BTK inhibitor
Renal function	General clinical variable	Influences TLS risk and feasibility of venetoclax initiation	Must be interpreted together with tumor burden
Infectious/immune vulnerability	Baseline patient and disease factor	May modify treatment morbidity and supportive-care needs	Frequently incompletely captured in retrospective datasets
Frailty/functional reserve	Host vulnerability	May predict toxicity, discontinuation and treatment burden	No single frailty instrument is an established treatment selector
Patient preference	Shared decision-making variable	Can change the preferred efficacy–toxicity–duration trade-off	Should be measured, not inferred from demographics

**Table 2:** Treatment characteristics relevant to individualized CLL therapy.

Domain	BTK Inhibitor-Based Strategy	Venetoclax-Based Fixed-Duration Strategy	Individualization Question
Duration	Often continuous as monotherapy	Defined duration	How strongly does the patient value treatment-free time?
Cardiovascular	Atrial fibrillation (AF), hypertension and other cardiac events require consideration; risk differs among individual BTK inhibitor	Different cardiovascular profile	What is the patient’s baseline cardiovascular vulnerability?
Bleeding	Relevant, particularly with anticoagulant or antiplatelet therapy	No BTK inhibitor-related platelet effect	Is clinically relevant bleeding risk present?
Renal/tumor lysis syndrome (TLS)	No venetoclax-style TLS ramp-up	Renal function and tumor burden influence TLS risk	Is initiation safe and practically feasible?
Cytopenia/infection	Relevant during prolonged therapy	Neutropenia and infection may be relevant, particularly in combinations	What is baseline infectious vulnerability?
Drug interactions	Medication review required	Medication review required, including cytochrome P450 3A (CYP3A) interactions	Can interacting chronic medication be modified safely?
Monitoring	Ongoing surveillance	More intensive during venetoclax initiation	Can the required monitoring be delivered reliably?
Treatment burden	Long-term adherence and exposure	Greater initial complexity but finite therapy	Which burden is more acceptable to the patient?
Preference sensitivity	Continuous disease suppression may be attractive to some patients	Treatment-free interval may be highly valued by others	What outcome does this patient value most?

Table 2 provides a clinical comparison rather than a hierarchy of treatment options. The importance of each characteristic varies between patients, and clinically relevant differences also exist between individual agents within the same drug class.

**Table 3:** CLL-specific decision contexts and clinically relevant treatment comparisons (illustrative; not prescriptive).

Decision Setting	Candidate Strategies to Compare	Required Modifiers/Estimand Limits
Untreated, TP53-intact	Continuous second-generation covalent BTK inhibitor (BTKi) vs. eligible fixed-duration venetoclax combinations	Cardiovascular/bleeding risk; TLS renal and nodal burden; monitoring capacity; planned treatment-free time
Untreated, TP53-disrupted	Guideline-supported BTKi-containing strategies; consider fixed duration only in appropriately supported contexts	Durability evidence by subgroup, TP53-specific risks and clinical suitability; no assumption of class equivalence
Relapsed after venetoclax	Covalent BTKi if appropriate and not previously resistant; other guideline-supported sequence	Prior response interval, tolerability, resistance, drug interactions and other salvage options
Relapsed after covalent BTKi	Venetoclax-based therapy if appropriate; noncovalent BTKi according to indication/exposure	Reason for BTKi cessation (intolerance vs. resistance); TLS; past BCL2 inhibitor (BCL2i) exposure
Dual-exposed/late-line	Guideline- and access-constrained later-line options; clinical trials where appropriate	Resistance, fitness, bridging/logistics, survival and quality-of-life priorities
Longitudinal reassessment	Continue, hold, switch or sequence only at defined new decision time	Time-dependent toxicity, progression, MRD where validated, prior exposures and immortal-time bias

**Table 5:** Minimum requirements for clinically trustworthy AI-assisted treatment selection in CLL.

Requirement	Why It Matters
Contemporary treatment cohorts	Historical effects may not generalize to current CLL therapy
Explicit treatment alternatives	Individualized selection requires a defined causal comparison
Separation of prognostic and treatment-effect objectives	High prognostic accuracy does not demonstrate comparative benefit
Treatment-specific efficacy endpoints	Benefit must be estimated for each relevant strategy
Clinically meaningful toxicity endpoints	Treatment choice depends on harm as well as disease control
Causal framework	Observational treatment assignment creates confounding by indication
Patient-preference data	Benefit–risk trade-offs differ between individuals
Transparent missing-data strategy	Missingness may reflect clinical practice and introduce bias
Calibration	Absolute predicted risks must correspond to observed risks
External validation	Performance must generalize beyond the development cohort
Temporal validation	Rapid evolution of CLL therapy can produce model drift
Explainability	Clinicians need to understand clinically important prediction drivers
Uncertainty estimation	The model should identify unreliable predictions
Ability to abstain	A recommendation should not be forced when evidence is insufficient
Prospective clinical evaluation	Statistical performance alone does not demonstrate clinical utility
Decision-curve/utility analysis	Improved prediction should translate into better decisions
Human factors and workflow evaluation	A useful model must function safely in real clinical practice
Human oversight	Final treatment choice remains a clinician–patient decision

**Table 6:** Relationship of the proposed CLL framework to existing hematology/oncology decision-support and trustworthy-AI approaches.

Framework/Evidence	Established Contribution	What Can Be Transferred	CLL-Specific Additional Requirement
CLL-TIM and CLL ML [97]	Infection/need prediction, disease risk	CLL-relevant data and individualized prognostic estimates	No comparative regimen-specific CATE or preference dialogue
Precision molecular tumor boards [98]	Genomic actionability, evidence-ranked eligible therapies	Evidence review and clinical sequencing context	No automatically calibrated absolute causal contrasts across regimens
Virtual molecular tumor board [99]	Evidence/rule-based therapeutic matching	Operational evidence scoring and history filters	Rules are not treatment-effect estimates; values/uncertainty not fully integrated
Hematology multi-criteria framework [100]	Explicit trade-offs in myeloma treatment	Transferable transparent criteria and patient relevance	Preference weights alone do not identify clinical causal effects
Oncology clinical decision-support (CDS) evaluations [12,15]	Guideline adherence, clinical workflow and safety	Prospective process-utility outcomes	Not a CLL counterfactual drug comparison engine
TRIPOD + AI/PROBAST + AI [13,94,95]	Reporting/critical appraisal of predictions	Calibration, design and bias assessment	Not an intervention or drug-selection model
DECIDE-AI/SPIRIT-AI/CONSORT-AI [13,14,15]	Early deployment and prospective AI-trial reporting	Human factors, trials and workflow evaluation	Standards rather than clinical algorithms
FUTURE-AI/STANDING Together [14,92]	Fairness, uncertainty, governance, dataset quality	Safeguards against drift, gaps and subgroup harm	No CLL-specific causal estimand
Present conceptual CLL architecture	Eligibility → CATE → audited risks → preference filters	Explicit comparator/time zero, abstention and outcomes protocol	Not implemented, estimated, externally validated or clinically tested

**Table 7:** Minimum methodological audit of mapped CLL AI/ML applications; findings distinguish reported evidence from unverified procedures.

Audit Domain	Evidence Indicated by Mapped Sources	Requirement for Trustworthy CLL Treatment Selection
Sample size/events/complexity	4149 CLL patients in CLL-TIM [5]; 109 in Meiseles [6]; mixed-diagnosis cohorts may be larger [40,45]	Report actual event counts, continuous degrees of freedom, learning complexity, and optimism; no generic 10 events-per-variable (EPV) rule
Internal evaluation	Hold-out testing documented in Zhao, Aoki and Bazinet [40,42,45]	Ensure patient-level split, nested tuning and confidence intervals; resampled test sets not independent external data
External/temporal validation	CLL-TIM includes validation [5]; Seheult evaluates real laboratory deployment [47]	Independent sites and eras essential for regimen-policy transport; lab validation is not treatment-policy validation
Missing data	Source [9] identifies incomplete reporting across historical literature	Report missingness by variable, center and treatment; impute within training folds; missing-not-at-random (MNAR) sensitivity analysis
Calibration & drift	Papangelou includes uncertainty [49]; Seheult reports data-drift monitoring [47]	Require intercept/slope/curve and temporal subgroup reassessment for clinical absolute risks
Overfitting & optimism	Small selected cohorts and high-dimensional genomics appear in source [9]	Bootstrap optimism correction, shrinkage, regularization and prespecified effect modifiers
Leakage/timing	MRD samples, clinical follow-up and longitudinal lab features create potential leakage channels	Index-time-only predictors; no post-index values, patient crossover between train/test, or immortal time
Decision utility/CATE	No mapped study verifies a deployed, multi-regimen CLL clinical CATE policy	Prospective comparison against usual care; harms, discontinuation, preferences and patient-level coverage

## Data Availability

No individual-patient clinical data were collected or analyzed for this narrative review. The focused screening is based on the publicly indexed PubMed records; the screening decisions can be supplied by the authors as an accompanying literature-screening log.

## Abbreviations

The following abbreviations are used in this manuscript:

AF	Atrial fibrillation
AI	Artificial intelligence
AUC	Area under the receiver operating characteristic curve
BCL2	B-cell lymphoma 2
BTK	Bruton tyrosine kinase
CLL	Chronic lymphocytic leukemia
CLL-TIM	Chronic Lymphocytic Leukemia Treatment-Infection Model
EHA	European Hematology Association
FISH	Fluorescence in situ hybridization
IGHV	Immunoglobulin heavy-chain variable region
MRD	Measurable residual disease
PFS	Progression-free survival
SLL	Small lymphocytic lymphoma
TLS	Tumor lysis syndrome
CATE	Conditional average treatment effect
IPTW	Inverse probability of treatment weighting
NLP	Natural language processing
SHAP	SHapley Additive exPlanations
XAI	Explainable artificial intelligence
EHR	Electronic health record
DCA	Decision-curve analysis
CNN	Convolutional neural network
C-index	Concordance index
ML	Machine learning
OS	Overall survival
ECG	Electrocardiography
PROBAST + AI	Prediction model Risk Of Bias ASsessment Tool + AI
